# Latent profiles of environmental awareness in Chinese college students: effects of psychological capital and digital engagement

**DOI:** 10.3389/fpsyg.2026.1758155

**Published:** 2026-05-14

**Authors:** Yi Liu, Lingeng Lu, Yongcheng Yao

**Affiliations:** 1 Department of Statistics, Zhengzhou Normal University, Zhengzhou, Henan, China; 2 Department of Chronic Disease Epidemiology, Yale School of Public Health, School of Medicine, Yale University, New Haven, CT, United States

**Keywords:** college students, environmental awareness, latent profile analysis, New Ecological Paradigm, psychological capital

## Abstract

**Introduction:**

This study aimed to identify distinct environmental awareness profiles among Chinese university students through latent profile analysis (LPA) and to examine how psychological capital and sociocultural factors predict the profile membership.

**Methods:**

Cross-sectional data from 1,107 undergraduates (73% female; average age of 20 years old) were collected using validated Chinese versions of the Psychological Capital Questionnaire (PCQ-26) and New Ecological Paradigm Scale (NEP-15) via the Questionnaire Star platform in Zhengzhou, China. LPA classified respondents based on 15 valid items of the NEP scale, followed by binary logistic regression analyzing demographic/psychological predictors for environmental awareness profiles.

**Results:**

LPA identified two latent profiles of environmental awareness: Low (Environmentally unengaged, 49.5%) and High (Holistically engaged, 50.5%), with no severe common method bias in the study data. Logistic regression showed that male students had a significantly lower probability of high awareness than females (OR = 0.519, 95%CI: 0.389–0.693); students with leadership roles had a higher probability of high environmental awareness (OR = 1.348, 95%CI: 1.043–1.743); daily internet usage hours (OR = 2.158, 95%CI: 1.742–2.672) and psychological capital (OR = 1.018, 95%CI: 1.012–1.024, a 1.8% increase per unit) were significantly positive predictors of high environmental awareness. Birthplace had no significant effect on profile membership.

**Conclusions:**

Environmental awareness heterogeneity in university students in China reflects synergistic influences of demographic traits, behavioral patterns (daily internet use), psychological resources (PsyCap), and social roles (leadership with positive effects) in environmental awareness. Environmental-related digital exposure and psychological capital are key intervention points for improving college students' environmental awareness.

## Highlights

- Two distinct environmental awareness profiles were classified in Chinese students using LPA.
- Female students, student leaders, longer daily internet use, and higher PsyCap predicted high environmental awareness.
- Digital platforms and PsyCap cultivation improved pro-environmental engagement.

## Introduction

The accelerating crises of climate change, biodiversity loss, and ecosystem degradation underscore the urgent need to cultivate robust environmental awareness, particularly among populations poised to shape future policies, economies, and societal norms (IPCC, 2023; Taylor, 2025; Shin et al., 2022; United Nation, 2025). As future leaders and decision-makers, university students represent a pivotal demographic member for advancing global sustainability agendas (Abo-Khalil, 2024; Filho et al., 2024; Suklun and Bengu, 2024; Zamora-Polo et al., 2019). The level of their environmental awareness not only influences their daily eco-friendly behaviors but also plays a significant role in advancing broader environmental protection efforts and the effectiveness of ecological civilization construction. Moreover, the formative nature of higher education offers a unique window for instilling lasting ecological values and competencies aligned with national and global sustainability goals, such as China's vision of “ecological civilization” (Wang et al., 2020). Despite their strategic importance, however, studies revealed significant gaps in youth engagement, with many students perceiving environmental issues as abstract or irrelevant to their daily lives (Zeeshan et al., 2021; Bayati, 2023). This gap between global urgency and individual disconnection highlights the pressing need to understand psychological and sociocultural drivers that foster or hinder environmental awareness in higher education settings.

Environmental awareness is a comprehensive reflection of an individual's cognitive, understanding, affective concern and behavioral inclinations toward environmental issues, serving as a fundamental driver of pro-environmental action practices (Kollmuss and Agyeman, 2002; Uzzell, 2000; Stern, 2000). Rooted in positive psychology, psychological capital (PsyCap) is composed of four core resources: self-efficacy, hope, resilience, and optimism (Luthans and Broad, 2022; Luthans and Youssef-Morgan, 2017). It has been linked to greater adaptability and goal pursuit (Carter and Youssef-Morgan, 2022; Culbertson et al., 2010). Recent studies suggest that PsyCap enhanced an individual's sense of social responsibility and engagement in prosocial behaviors, including environmental stewardship (Albrecht et al., 2023; Chipfupa et al., 2021). For example, students with high self-efficacy were more likely to believe in their personal capacity to mitigate climate impacts (Bartlett et al., 2022; Turcotte-Tremblay et al., 2024), while hopeful individuals demonstrated stronger long-term commitment to environmental goals (Malboeuf-Hurtubise et al., 2024; Geiger et al., 2023). These findings position PsyCap as a potentially significant antecedent of environmental awareness. Theoretically, this link can be understood through a prosocial lens: PsyCap cultivates psychological resources that predispose individuals to look beyond self-interest, fostering a sense of connection with others and with the broader social and ecological world. This expanded sense of responsibility in turn motivates attention to and concern for environmental issues. However, the direct relationship between PsyCap and environmental awareness remains underexplored, particularly in non-Western educational context.

Current research on the relationship between university students' environmental awareness and psychological capital remains insufficient, and most existing studies treated environmental awareness as a unidimensional variable, overlooking potential heterogeneity within the group (Cruz and Manata, 2020; Torres et al., 2023). In addition, existing research predominantly focused on Western contexts (Cruz and Manata, 2020; Torres et al., 2023; Mavisakalyan and Tarverdi, 2019), neglecting Global South perspectives (e.g., China's rapid urbanization and educational reforms), which may shape environmental consciousness in distinct ways. To fill these gaps, we adopted a person-centered approach, latent profile analysis (LPA), which offers a more nuanced alternative to traditional variable-centered methods. Rather than relying on arbitrary cutoffs (e.g., median or tertile splits) or assuming population homogeneity, LPA empirically identifies distinct latent subgroups based on their response patterns across multiple indicators. LPA classifies everyone into a group based on the probability of membership. The New Ecological Paradigm (NEP) scale (Dunlap et al., 2000; Luthans et al., 2007), which assesses five interrelated dimensions (limits to growth, anti-anthropocentrism, fragility of nature's balance, rejection of human exceptionalism, and ecological crisis awareness). The scale measures an individual's ecological worldview of fundamental beliefs about humanity's relationship with the natural environment: whether humans are above nature or nature is fragile and interconnected with human life. It provides a theoretically grounded basis for profiling individuals' environmental awareness, predicting their environmental attitudes and potential behavior changes. While prior studies have typically used the NEP as a composite sum score, its five dimensions may not develop uniformly across individuals. Theoretically, the NEP scale of ecological worldview is a multifaceted construct, and different facets may be endorsed independently depending on an individual's experience, education and cultural context. Empirically, recent methodological advances in person-centered analyses supported examining its dimensions as potentially asynchronous indicators, enabling the analysis of qualitative differences (e.g., profile shapes) beyond mere differences in intensity levels (Rhead et al., 2018). Thus, LPA is particularly suited to examining whether such heterogeneous patterns exist in our Chinese university sample, rather than assuming a priori that all individuals can be appropriately characterized by a single sum score.

Previous studies on environmental awareness are mainly from Western societies (Schultz et al., 2005; Krettenauer, 2017; Wild and Schulze Heuling, 2024), often overlooking the unique sociocultural, institutional and developmental contexts of the Global South. In countries such as China, where rapid urbanization, educational transformation, and policy-driven sustainability initiatives are reshaping young individual's lived experience, the interplay between PsyCap, digital engagement, and sociodemographic factors (e.g., sex, student leadership roles) may influence environmental awareness via distinct ways. For instance, digital platforms increasingly mediated environmental information access and activism among youth (Xie et al., 2024; Li et al., 2025), while leadership experience in campus organizations may have complex effects on environmental agency (Mohamed et al., 2025). Sex differences in environmental concern and behavior have also been documented globally (Dhenge et al., 2022; Milfont and Sibley, 2016). However, these contextual variables remain under-integrated into theoretical models of environmental awareness in Chinese student populations.

To address these gaps, we conducted a cross-sectional study of Chinese college students to identify distinct latent profiles of environmental awareness using the valid items of the NEP scale, to examine how PsyCap, digital engagement, sex, and leadership status differentially predicted profile membership and to explore the implications for theory and practice in sustainability education. We hypothesized that university students' psychological capital might be intrinsically linked to their environmental awareness. For student leadership, we explored its potential effect without a directional hypothesis. The findings will offer theoretical insights into the formation mechanisms of university students' environmental awareness while providing scientific references and decision-making support for universities to develop targeted environmental education among college students in the Global South. Such initiatives aim to enhance students' environmental awareness and positive psychological resources, thereby cultivating the next generation of sustainability leaders and contributing to ecological civilization efforts.

## Participants and methods

### Study participants

In May 2023, an online survey was conducted via the “Questionnaire Star” platform to examine the environmental awareness and risk factor of Chinese university students using a convenience sampling approach. The research team first contacted academic advisors to explain the study's objectives. Questionnaires were then distributed to all students in the participating classes via WeChat class group. All students were free to choose whether to participate, all questionnaires were anonymously answered. The study was approved by the Ethics Committee of Zhengzhou Normal University (ZZNU-2023-006). A total of 1,107 undergraduate students voluntarily participated and provided valid responses. Among them, 299 (27.0%) were male, and 808 (73.0%) were female, aged 18–26 (mean: 20 years). Basic demographic data in the questionnaire included sex, age, student leadership role, birthplace, and daily internet usage hour(s) (as a proxy of digital engagement). All participants provided online written informed consent when they were administered the questionnaires.

### Psychological Capital Questionnaire (PCQ)

The PCQ (24 items) was developed by (Luthans and Broad 2022); (Luthans and Youssef-Morgan 2017), and its Chinese version with 26 items by Zhang Kuo (Zhang et al., 2010) was used to comprehensively assess students' positive psychology. The 26-item scale measures four dimensions—self-efficacy, hope, resilience, and optimism. Responses were recorded on a seven-point Likert scale (1 = “strongly disagree,” 7 = “strongly agree”), with higher scores indicating higher levels of positive psychology. The scale's Cronbach's α coefficient in this study was 0.920. The fit indices were RMSEA = 0.075 [90%CI (0.072, 0.078), *P* < 0.001], CFI = 0.820, TLI = 0.801, and SRMR = 0.081, indicating that the validity of PCQ was acceptable.

### NEP questionnaire

The NEP questionnaire, developed by (Dunlap et al. 2000) and (Luthans et al. 2007) and adapted into Chinese by (Wu et al. 2012), was used to measures individuals' ecological values and environmental awareness. It assesses how individuals perceive the relationship between humans and nature, reflecting different stages of ecological literacy. Different needs of environmental education depend on individuals' perception and acceptance of environmental education at different stages. A two-factor model has previously been shown for the 15-item scale (Manoli et al., 2019). Responses were recorded on a five-point Likert scale (1 = “strongly disagree,” 5 = “strongly agree”), with higher scores indicating greater environmental concern. The fit indices were RMSEA = 0.098 [90%CI (0.091, 0.105), *P* < 0.001], CFI = 0.917, TLI = 0.897, and SRMR = 0.047, indicating that the validity of 15-item NEP scale was acceptable. The scale's Cronbach's α coefficient in this study was 0.792.

### Statistical analysis

LPA was conducted using Mplus 8.3. The 15 valid NEP item scores served as observed variables, and models with 1–5 profiles were sequentially tested. The final model's fit was evaluated using three criteria: (1) Information criteria: Akaike Information Criterion (AIC), Bayesian Information Criterion (BIC), and adjusted BIC (aBIC) were used to compare expected vs. observed values, with smaller values indicating better fit. (2) Classification accuracy: Entropy (range: 0–1) assessed classification uncertainty, with values closer to 1 (>0.70) indicating that individuals were classified with high certainty into their respective latent classes. (3) Likelihood ratio tests: The Lo-Mendell-Rubin adjusted likelihood ratio test (LMR) and bootstrap likelihood ratio test (BLRT) compared fit between *k*−1 and *k*-class models. A *P* < 0.05 for LMR and BLRT indicated that the *k*-class model was superior. While these criteria guided the model selection, the best model in LPA was practically selected based on the combination of the lowest information criteria values, a statistically significant likelihood ratio and a high entropy value, which indicates good classification certainty. In addition, interpretability and minimum class size (proportion >5%) were also in consideration in determining the optimal profile solution. Unlike studies relying solely on total scores, LPA accounts for intra-group heterogeneity, avoiding potential misclassification where individuals with identical total scores may differ in item-level responses.

SPSS 27.0 was used for additional analyses. Normally distributed continuous data (skewness and kurtosis absolute values < 1) were described as mean ± standard deviation. Categorical data were presented as frequencies and percentages, with chi-square tests for group comparisons. Variables significant in univariate analyses were included as predictors in binary logistic regression, with latent profiles as the outcome. Multicollinearity test was also performed. Continuous variables including psychological capital were mean-centered for the logistic regression. A two-tailed *P* < 0.05 was considered statistically significant.

## Results

### Common method bias test

Harman's single-factor test was used to assess common method bias. The results showed that there were six factors with eigenvalues greater than 1, and the first factor explained 26.1% of the variance (below the critical threshold of 40%), indicating that no severe common method bias existed in the study data.

### Latent profile analysis of university students' environmental awareness

The total environmental awareness score of university students was 3.44 ± 0.47.

Latent profile models with 1–5 profiles were sequentially tested (Table 1). As the number of classes increased, the information criteria (AIC, BIC, and aBIC) decreased. The two-class model demonstrated a significant LMR test result (*P* < 0.05) and the entropy value is above 0.7. The three-class model showed better entropy and information criteria but was rejected due to non-significant LMR test and a tiny class proportion (< 5%), which lacks practical interpretability. Thus, the two-class model was selected as the best-fitting solution for university students' environmental awareness. The proportions of two profiles were 49.5% (named as Environmentally unengaged or Low awareness) and 50.5% (Holistically engaged or High awareness), respectively. To simplify the expression, hereafter we coined two groups as low and high awareness.

**Table 1:** Model fit indices for latent profile analyses of environmental awareness in university students (*n* = 1,107).

Model	AIC	BIC	aBIC	Entropy	LMR	BLRT	Profile proportions
1	44,747.104	44,897.386	44,802.099				
2	42,450.663	42,681.095	42,534.988	0.857	0.000	0.000	0.50/0.50
3	41,492.524	41,803.107	41,606.180	0.913	0.050	0.000	0.03/0.48/0.49
4	40,829.708	41,220.442	40,972.695	0.920	0.276	0.000	0.40/0.01/0.09/0.50
5	40,308.009	40,778.894	40,480.326	0.891	0.155	0.000	0.02/0.33/0.08/0.42/0.15

**Figure 1:**
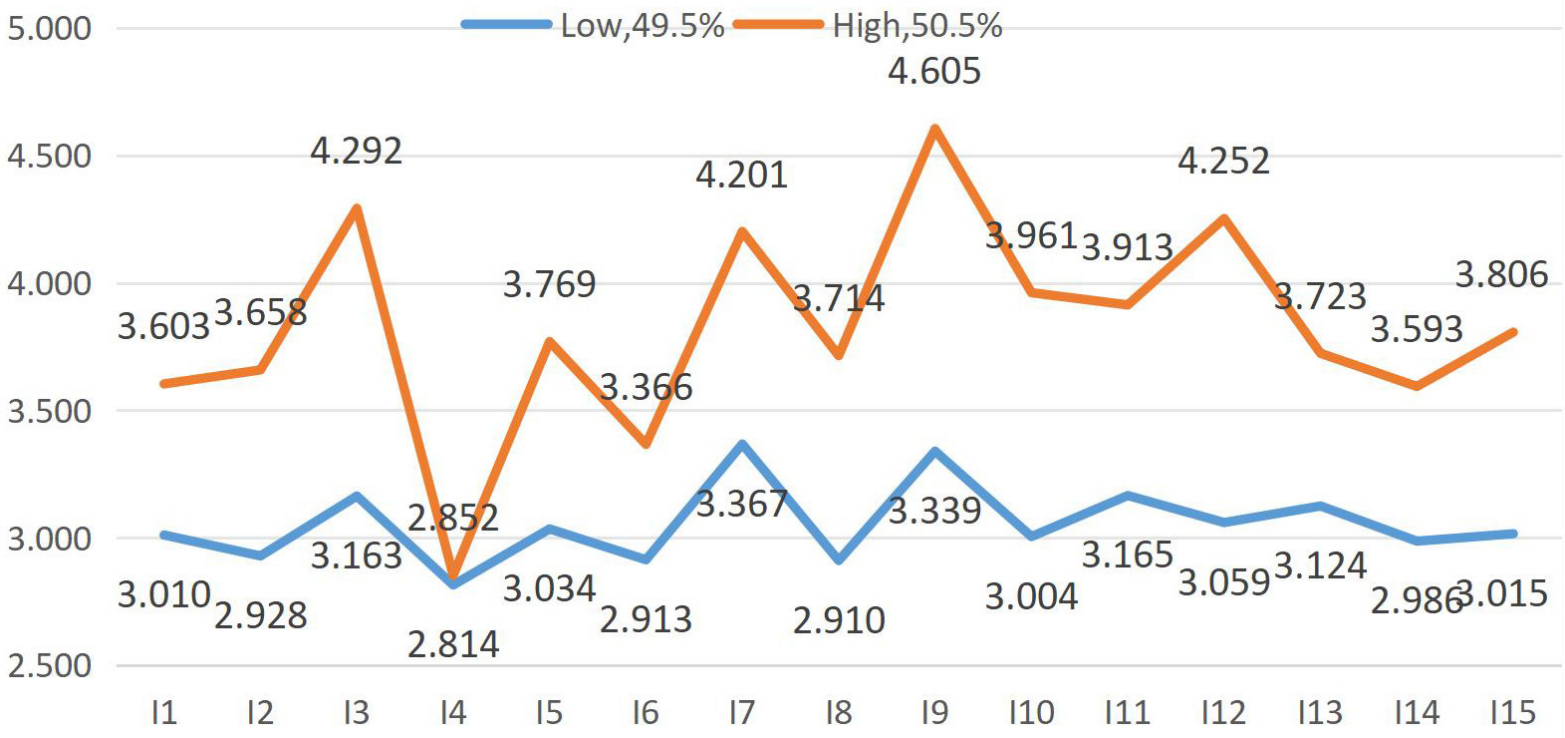
The average value of each item score of the two potential categories.

Figure 1 presents the conditional mean plots of the two latent profiles across the 15 items of environmental awareness. Higher scores correspond to greater environmental awareness according to the scale's theoretical framework. Based on the characteristic patterns of conditional means for each latent profile (Low–High) in these items, we assigned the following profiles: Low Awareness group (49.5%). Individuals in this profile demonstrated relatively low scores across all measurement items, indicating a lower overall level of environmental consciousness. High Awareness group (50.5%). Individuals in this class achieved consistently high scores across almost all measurement items, reflecting an advanced level of environmental consciousness and commitment.

### Demographic distribution across latent profiles

Chi-square tests including for proportion trend were conducted to examine demographic differences and their proportion trend among the latent profiles Significant difference and trend (*P* < 0.01) were found in sex, student leadership role, and daily internet usage time, but not in birthplace (*P* > 0.05). Male students were underrepresented in the High Awareness group, but this result may be biased by the severely gender-imbalanced sample (73% female). Student leaders were more prevalent in the High Awareness group than in the Low Awareness group. The Low Awareness group had a higher proportion of students with < 1 h of daily internet usage and a lower proportion of those with >4 h compared to the High Awareness group (Table 2).

**Table 2:** Comparison of two profiles' scores among different characteristics [*n* (%)].

Variable	Low (*n* = 548)	High (*n* = 559)	*P*_trend_	*P*
Sex				
Male	193 (35.2)	106 (19.0)	1.14 × 10^−9^	1.13 × 10^−9^
Female	355 (64.8)	453 (81.0)		
Leadership				
Yes	295 (53.8)	363 (64.9)	1.69 × 10^−4^	1.68 × 10^−4^
No	253 (46.2)	196 (35.1)		
Daily internet usage(hours)				
< 1 h	70 (12.8)	10 (1.8)	1.69 × 10^−14^	7.87 × 10^−15^
1–4 h	191 (34.9)	156 (27.9)		
>4 h	287 (52.4)	393 (70.3)		
Birth place				
Urban	127 (23.2)	147 (26.3)	0.25	0.48
Suburban	104 (19.0)	104 (18.6)		
Rural	317 (57.8)	308 (55.1)		

### Multivariate logistic analysis of latent profiles

A logistic regression was performed with the latent profiles as the outcome and the predictors of sex, student leadership role, daily internet usage, and psychological capital. Multicollinearity diagnostics showed that all variance inflation factors (VIF) ranged from 1.02 to 1.04 (below the critical value of 10), indicating no multicollinearity existed.

Compared with female students, males had a significantly lower probability of high environmental awareness (OR = 0.519, 95%CI: 0.389–0.693, and *P* = 8.00 × 10^−6^). Compared with those without leadership, Students with leadership had a significantly higher probability of high environmental awareness than those without (OR = 1.348, 95%CI: 1.043–1.743, and *P* = 0.023). As the daily online time increased, the probability of having a high level of environmental awareness showed a significant increase (*P*_trend_ = 1.86 × 10^−12^, OR = 2.158). Students with high psychological capital had a significantly higher probability of environmental awareness than those with low one (OR = 1.018, 95%CI: 1.012–1.024, and *P* = 2.81 × 10^−8^) (Table 3).

**Table 3:** Logistic regression of different characteristics and psychological capital on environmental awareness profiles (High vs. Low).

Variable	β	SE	Wald	*P*_trend_	OR	95%CI
Sex						
Male	−0.655	0.147	19.851	8.00 × 10^−6^	0.519	0.389–0.693
Female	Reference				Reference	
Leadership						
Yes	0.299	0.131	5.198	0.023	1.348	1.043–1.743
No	Reference				Reference	
Daily internet usage (hours)	0.769	0.109	49.626	1.86 × 10^−12^	2.158	1.742–2.672
Psychological capital	0.018	0.003	30.832	2.81 × 10^−8^	1.018	1.012–1.024

## Discussion

Traditional approaches to assessing environmental awareness often treat it as a unidimensional trait (Dunlap et al., 2000), potentially obscuring meaningful heterogeneity in how individuals relate to ecological issue (Cruz and Manata, 2020; Torres et al., 2023; Zhang et al., 2024). While the NEP scale (Dunlap et al., 2000) measures five dimensions (e.g., growth limits, anthropocentrism, nature balance, human exceptionalism, and ecological crisis), it is typically aggregated into a single score. In contrast, person-centered methods such as LPA allow us to identify subgroups with distinct configurations across these dimensions. In this study, we employed person-centered LPA to examine environmental awareness in a sample of Chinese college students in Zhengzhou and identified two latent profiles: Low (49.5%) and High (50.5%) environmental awareness. This study moves beyond total scores to reveal the latent subgroups of NEP endorsement in this population. Critically, these profiles differed primarily in their overall level of endorsement across all fifteen NEP items, rather than in qualitative patterns. This result demonstrated that university students might exhibit significant differences in environmental cognition, attitudes, and behavioral intentions. The finding indicates that environmental education cannot adopt a one-size-fits-all approach, precision environmental education intervention should be designed to promote environmental sustainability.

Students in the Low Awareness group scored low across all NEP items, particularly in ecological crisis perception (mean scores range 2.814–3.367). This finding indicates that these students lack sufficient understanding of the severity and urgency of current ecological issues (such as climate change, biodiversity loss, and pollution) and perceive environmental crises as “distant from themselves,” failing to recognize the direct connection between environmental problems and their personal lives. This aligns with prior research showing that many young adults perceived ecological issues as abstract or geographically/temporally distant (Bayati, 2023). Given that this group constitutes nearly half of the sample, the findings underscore a persistent gap in environmental literacy among Chinese university students in Zhengzhou. The lack of substantive environmental education courses in schools fails to stimulate students' deep reflection on environmental issues, resulting in lower levels of environmental awareness. The large size of this group serves as a warning that improving university students' environmental awareness remains a long-term challenge, requiring multichannel efforts to strengthen environmental education and advocacy, thereby enhancing students' understanding of and responsibility toward environmental issues.

The High Awareness group accounts for the majority (50.5%) of the sample, with consistently high scores across all 15 NEP items (mean scores range 2.852–4.605), reflecting an advanced level of environmental consciousness and a strong sense of environmental responsibility. This group is the core force of environmental advocacy on campus, and their high awareness may stem from frequent exposure to environmental information via digital platforms, strong psychological capital, or personal attention to ecological issues. Schools should leverage the demonstrative effect of this group, encourage them to carry out peer environmental education activities (e.g., environmental sharing sessions, waste sorting promotion), and further expand the influence of environmental protection on campus by building a positive environmental protection atmosphere.

Several sociodemographic and psychological factors were significantly associated with profile membership. Female students were more likely to belong to the High Awareness group, a finding consistent with decades of environmental psychological research linking sex to environmental concern (Echavarren, 2023; Li et al., 2022). This pattern is often explained via gendered socialization: women are more inclined toward prosocial behavior, care-oriented ethics and societal expectations (Mavisakalyan and Tarverdi, 2019). Sociological theory posits that women's socialization process emphasizes an “ethics of care,” making them more emotionally attuned to public welfare issues like the environment. Additionally, environmental actions are often perceived as “soft responsibilities,” aligning with societal expectations of women, whereas men may prioritize “hard” issues such as economics and technology, diverting attention from environmental concerns. Psychological research indicates that women generally exhibit higher emotional engagement with public issues (e.g., environmental concerns), and this stereotype may motivate them to pay more attention to environmental topics (Fischer et al., 2018; Chaplin, 2015). Moreover, women tend to build group identity through cooperative behaviors (e.g., environmental advocacy), thereby enhancing their environmental concern. While we do not claim causality, this association reinforces the importance of considering sex in the design of inclusive environmental education programs.

Similarly, holding a student leadership role was a significant predictor of high awareness, highlighting the role of organizational responsibility in boosting environmental awareness (He et al., 2023; Ting et al., 2024). This may stem from two factors: one is role-modeling effect. Student leaders, as “servants” of their peers, are more likely to internalize environmental responsibility through exemplary behaviors. Another is exposure to policies. Involvement in class management increases access to school-led environmental initiatives (e.g., energy-saving campaigns, waste-sorting supervision), creating a feedback loop of role obligation, behavioral practice and then attitude reinforcement in sequence.

Daily internet usage hours as a measure of digital engagement here were positively associated with higher awareness. While this metric is admittedly rough, representing a methodological limitation, it reflects a broader trend: digital platforms serve as critical spaces for environmental information dissemination, discourse, and mobilization among youth (Xie et al., 2024; Li et al., 2025). In this study, we found students who spent more time in digital media had high environmental awareness. In contrast, the proportion of students who spent less than 1 h online per day in the Low awareness group was the highest. This finding also suggests that in the digital age, social media and short video platforms are an important carrier for the dissemination of environmental issues (such as environmental bloggers' popular science and ecological disaster news) to increase individuals' environmental awareness by exposing students to the knowledge and debate of climate change and crisis (Lowan-Trudeau, 2023; Hafferty et al., 2024). Through in-depth environmental protection content (such as the documentary “Planet Earth,” environmental NGO initiatives, and policy interpretation articles), further participating in environmental protection communities (such as Douban's “Zero Waste Life” group and Weibo environmental protection topic discussions), individuals' environmental awareness is enhanced. However, this study did not distinguish between content types that whether they are environment-related information, limiting causal interpretation. Thus, the significant association of internet use and profile membership in this study should be interpreted cautiously. Further studies should employ more granular measures (e.g., frequency of exposure to environmental content, engagement depth) to clarify this relationship.

Notably, we found that students with high psychological capital were more likely to believe that their life behavior could change the current environmental situation. This belief could bridge awareness to action, being pro-environmental activists. They believed in personal impact on environmental changes, driving sustained pro-environmental behavior. For instance, individuals who believed that my action in garbage sorting had practical effects on the environment, helping energy regeneration and reducing pollution, were more likely to continue to participate in environmental protection practices. In contrast, those with low psychological capital were prone to feel powerless because they thought that environmental problems were complex and difficult to solve, reducing their willingness to pay attention. Individuals with a strong sense of psychological capital tend to concern future environmental conditions (Albrecht et al., 2023; Chen and Tang, 2022). This future perspective makes them willing to work for long-term environmental benefits, translating awareness into lasting commitments, such as participating in environmental volunteer activities and supporting sustainable consumption. Psychological research shows that a sense of psychological capital (e.g., hope) can enhance an individual's goal-oriented behavior and transform environmental concerns from short-term concerns to long-term commitments. Thus, PsyCap may function as a critical bridge between awareness and action, a mechanism particularly relevant in contexts like China, where top-down environmental policies require bottom-up citizen engagement for effective implementation (Wang et al., 2020).

There are some limitations in this study. First, the cross-sectional design limits causal inferences from the association of environmental awareness with demographic characteristics and psychological capital. In future studies, longitudinal or randomized controlled studies can be implemented to further explore the relationship between the two. Second, the data in this study were derived from self-report and participation was voluntary, which may have self-report and self-selection bias, although the scales in the used questionnaires have been proven to have good reliability and validity. Third, digital engagement was measured solely by daily internet usage hours, which did not capture content relevance, plate type or active vs. passive consumption. Further research should integrate media diaries or digital trace data to better assess online environmental exposure. Fourth, although our sample is relatively large and drawn from a key Chinese city, findings may not be able to be generalized to rural or less urbanized regions, given that the limitation of convenience sampling method and geographic representativeness. Fifth, the measurement of ecological worldview using the NEP scale, which assesses beliefs rather than behaviors, knowledge or values, may have some limitation in interpreting as comprehensive environmental awareness. It warrants further investigation to use the NEP scale developed in Western contexts to evaluate ecological worldview in non-Western settings. Sixth, while the latent profiles identified in this study were an empirical finding, which showed the NEP dimensions co-varied relatively uniformly, different profiles may exist in other populations. Finally, gender imbalance in this study and unmeasured confounders such as socioeconomic status, parental education, and prior environmental education may influence the observed association. Future studies should examine urban-rural disparity and other sociocultural moderators. Moreover, the finding in this study needs further validation in independent studies with a relatively large sample size.

## Conclusion

In this study, we found that 49.5% of Zhengzhou college students had low environmental awareness, and 50.5% had high environmental awareness. Although a little higher proportion of students had a high environment awareness, there were almost the half of students with low awareness, indicating that there is room for improvement in campus environmental education. Environmental education for college students needs to design targeted intervention strategies for the low awareness group, focusing on connecting environmental issues with daily life and improving basic environmental literacy, and at the same time leveraging the high awareness group to build a campus environmental protection ecology to drive the overall improvement of environmental awareness.

University students' environmental awareness was shaped by demographic traits, behavioral habits, psychological resources, and social roles: High awareness was associated with female, longer daily internet usage, and strong psychological capital, and student leadership. More daily internet usage might expose students to climate change debate, influencing their awareness.

These findings provide scientific evidence for precision-based environmental education interventions targeting college students. For the environmentally unengaged (low awareness) group, the core intervention strategy is to improve basic environmental literacy through compulsory coursework, on-site nature exposure programs and daily life-oriented awareness campaigns, which aim to establish fundamental ecological knowledge and connect environmental issues with students' daily lives. For the holistically engaged (high awareness) group, the focus is to leverage their demonstrative role: appoint them as campus environmental role models, support them in organizing peer education activities (e.g., environmental sharing sessions, waste sorting promotion) and build a peer network to amplify pro-environmental behaviors on campus.

## Data Availability

The raw data supporting the conclusions of this article will be made available by the authors, without undue reservation.
